# Vitamin D Status in Children with Primary Immunodeficiency Disorders Receiving Vitamin D Supplementation: A Retrospective Cross-Sectional Study

**DOI:** 10.3390/nu18172845

**Published:** 2026-08-31

**Authors:** Nel Dąbrowska-Leonik, Renata Grzywa-Czuba, Monika Wasiak, Mikołaj Wasil, Weronika Zaczek, Maria Żebrowska, Małgorzata Pac

**Affiliations:** 1 Department of Immunology, Children’s Memorial Health Institute, 04-730 Warsaw, Poland; 2 Department of Microbiology and Clinical Immunology, Children’s Memorial Health Institute, 04-730 Warsaw, Poland; r.grzywa@ipczd.pl; 3 Faculty of Medicine, Collegium Medicum, Cardinal Stefan Wyszynski University, 01-938 Warsaw, Poland; wasiakmonika.lo@gmail.com (M.W.); mikolaj.wasil@gmail.com (M.W.); weronikazaczek25@gmail.com (W.Z.); mariazebrowska03@gmail.com (M.Ż.)

**Keywords:** vitamin D, inborn error of immunodeficiency, children

## Abstract

**Background**: Vitamin D plays an important immunomodulatory role, and vitamin D deficiency has been associated with an increased risk of infections and autoimmune disorders. Although patients with some primary immunodeficiency disorders (PID) exhibit higher rates of vitamin deficiency, there are currently no evidence-based recommendations regarding whether children with PIDs require higher vitamin D supplementation than healthy peers. This study aimed to evaluate the nutritional status of vitamin D and vitamin D supplementation in children with PIDs. **Methods**: The study included 132 children diagnosed with PIDs according to the 2024 update of the phenotypic classification by the International Union of Immunological Societies (IUIS). The following variables were evaluated: body mass index (BMI), 25-hydroxyvitamin D (25(OH)D), calcium, phosphorus, immunoglobulins G, A and M, and number of neutrophils and lymphocytes in the blood. **Results:** Vitamin D deficiency occurred in 13% of all tested children, and in 22 and 33% of children with predominantly antibody deficiencies and complement deficiencies, respectively, with the median vitamin D supplementation of 1000 IU. The median serum 25(OH)D concentration was 30.75 ng/mL (32.7 ng/mL in girls and 30 ng/mL in boys). The multiple linear regression model showed that the concentration of serum phosphorus level (b = 0.312; *p* = 0.0497) and immunoglobulin G level (b = 0.433; *p* = 0.0009) had the greatest influence on the concentration of 25(OH)D. No statistically significant differences in 25(OH)D concentrations were observed among the different IUIS diagnostic groups or between children with normal and low IgG levels. **Conclusions**: In this retrospective cross-sectional study, children reportedly receiving median 1000 IU supplementation had median 25(OH)D of 30.75 ng/mL with 13% deficiency prevalence; however, causal attribution requires prospective evaluation.

## 1. Introduction

Vitamin D plays a critical immunomodulatory role by supporting both innate and adaptive immune functions. The active form of vitamin D interacts with the vitamin D receptor, which is expressed on most immune cells, including T and B lymphocytes, dendritic cells, and macrophages. This signaling promotes antimicrobial peptide production, enhances pathogen recognition, and modulates cytokine responses, thereby contributing to host defense against infections and regulating inflammation [1,2,3].

Children with primary immunodeficiency disorders (PID), currently referred to as inborn errors of immunity (IEI), are characterized by an increased risk for infections, autoinflammations, severe allergies, lymphoproliferations, autoimmune complications, and malignancy. PID are a heterogeneous group of diseases with the incidence of 6 per 10,000 people [4], resulting from pathogenic variants in more than 500 very rare genes that impair the function of the immune system. PID can be classified according to the mechanism of disorder development. According to the 2024 update of the International Union of Immunological Societies (IUIS) classification, PIDs (IEI) are categorized into 10 major groups based on the underlying immunological defect, including immunodeficiencies affecting cellular and humoral immunity, combined immunodeficiencies with associated or syndromic features, predominantly antibody deficiencies, diseases of immune dysregulation, congenital defects of phagocyte number or function, defects in intrinsic and immunity, autoinflammatory disorders, complement deficiencies, bone marrow failure, and phenocopies of inborn errors of immunity associated with autoantibodies or somatic variants [5].

Several observational studies and systematic reviews have demonstrated that vitamin D deficiency is common in patients with selected PIDs, particularly in those with ataxia telangiectasia syndrome (ATS) and common variable immunodeficiency (CVID) [6,7,8,9,10,11,12]. Treatments for IEI depend on clinical severity and the immune pathway impacted: patients may not require treatment, may be managed with prophylactic antibiotic therapy, immunoglobulin replacement therapy, immunosuppressive therapy including corticosteroids, cytokine inhibitors, mTOR (mechanistic target of rapamycin) inhibitors, JAK-STAT (Janus kinase-signal transducer and activator of transcription) inhibitors, or may require hematopoietic stem cell transplantation (HSCT), gene therapy to resolve genome abnormalities, or enzyme replacement therapy for metabolic disorders [13,14,15]. Maintaining adequate vitamin D status may therefore represent an important supportive measure for optimizing immune function and improving overall clinical outcomes in these patients.

The Endocrine Society recommends empiric vitamin D supplementation in children to prevent nutritional rickets and potentially reduce the risk of respiratory tract infections, which is particularly relevant for those with immunodeficiency [16,17,18,19,20]. The suggested dosage for children aged 1 to 18 years is daily intake through fortified foods, vitamin formulations, or supplements, with specific dosing individualized based on risk factors and serum 25-hydroxyvitamin D (25(OH)D) levels.

However, no professional society has developed specific recommendations regarding vitamin D supplementation for children with PIDs, despite their increased susceptibility to infections and the relatively high prevalence of vitamin D deficiency reported in several PID subgroups [1,21].

This study aimed to evaluate the nutritional status of vitamin D in children with PID during supplementation.

## 2. Materials and Methods

This retrospective cross-sectional study with data extraction included 132 patients diagnosed with PIDs out of 525 children who were hospitalized from 1 January 2023 to 31 October 2025 in the Department of Immunology, The Children’s Memorial Health Institute in Warsaw, Poland. Data collection was in November 2025. Statistical analysis in December 2025. Manuscript drafting in January 2026.

The inclusion criterion was a diagnosis of PID established according to the European Society for Immunodeficiencies (ESID) Registry—Working Definitions for Clinical Diagnosis of PID [22].

The exclusion criterion was hematopoietic stem cell transplantation (HSCT) before data collection.

The main limitation of the study design is its retrospective cross-sectional nature, which precludes causal interference. In addition, potential confounding factors, including disease severity, concomitant medications, gastrointestinal malabsorption, dietary habits, sun exposure, and individual differences in vitamin D metabolism, could not be fully controlled.

The study population consisted of 40 girls and 92 boys, ranging in age from 1 month to 17 years. Patients were classified according to the 2024 update of the International Union of Immunological Societies (IUIS) phenotypic classification into the following groups:

1. Immunodeficiencies affecting cellular and humoral immunity (*n* = 5).
2. Combined immunodeficiencies with associated or syndromic features (*n* = 34).
3. Predominantly antibody deficiencies (*n* = 40).
4. Diseases of immune dysregulation (*n* = 22).
5. Congenital defects of phagocyte number or function (*n* = 13).
6. Defects in intrinsic and innate immunity (*n* = 4).
7. Autoinflammatory disorders (*n* = 6).
8. Complement deficiencies (*n* = 8).

No patients were classified into bone marrow failure syndromes or phenocopies of inborn errors of immunity. Demographic data included sex and age. Detailed diagnoses within each IUIS category are presented in the Results section and Supplementary Table S1.

Vitamin D supplementation doses were obtained from medical records based on routine parental interviews. The reported dose represented the prescribed dose, constant for at least 3 months, used from the beginning of October to the end of April or throughout the year if skin synthesis in summer was insufficient; adherence was assessed by the attending physician. Various formulations and forms of vitamin D3 were administered.

The anthropometric data included the body mass index (BMI), height based on percentile charts according to the World Health Organization (WHO) AnthroPlus version 1.0.4.

**Figure 1:**
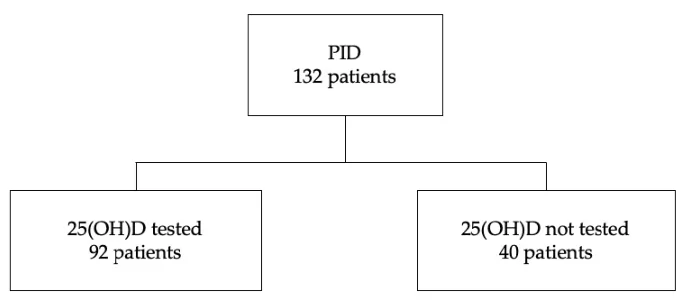
A study flow diagram illustrating patient selection, including the number of hospitalized children, eligible patients with primary immunodeficiency disorders, and those with available serum 25(OH)D measurements.

Serum total 25-hydroxyvitamin D concentration, including both 25(OH)D [(25(OH)D2 and 25(OH)D3)], was measured in 92 children by the attending physician according to patient-specific clinical indications during hospitalization, and the results were collected retrospectively (Figure 1). Patients with and without 25(OH)D determination did not differ in terms of age, sex, diagnosis or treatment.

Blood samples were collected by qualified nursing staff. The total 25(OH)D concentration was determined using the IDS-iSYS immunodiagnostic system and a method based on chemiluminescence technology (IDS 25 VitDs) (Immunodiagnostic System Ltd., Boldon, UK). The practical measurement range was from 4 ng/mL to 110 ng/mL, according to the IDS-iSYS system document provided for testing IDS 25 VitDs from the manufacturer. Vitamin D status was defined as deficiency (<20 ng/mL), suboptimal concentration (20–30 ng/mL), optimal concentration (30–50 ng/mL) and high concentration (50–100 ng/mL) [19].

Serum concentrations of immunoglobulin G (IgG), M (IgM), and A (IgA) were determined by nephelometry on automated BN ProSpec and Atellica^®^ NEPH 630 Siemens Healthineers (since 2019) using high-specificity monoclonal antibodies (Siemens Healthineers, Erlangen, Germany).

### Statistical Analysis

The data distribution was non-normal (Shapiro–Wilk normality test). Comparisons between groups were performed using the Chi-2 Pearson test for nominal data and Kruskal–Wallis test for quantitative variables. The relationships between 25(OH)D concentration and BMI percentile were evaluated using Spearman’s rank-order correlation coefficient. Furthermore, a multiple linear regression was also conducted to identify which of the examined parameters determine changes in 25(OH)D concentration.

All statistical analyses were carried out using the TIBCO Statistica version 13.3 software (TIBCO Software Inc., Santa Clara, CA, USA). Statistical significance was set at *p* < 0.05.

The study was conducted in accordance with the principles of the Declaration of Helsinki Guideline on Biomedical Research Involving Human Subjects and was approved by the Bioethics Committee at the Children’s Memorial Health Institute (Decision No. 59/KBE/2025).

## 3. Results

Vitamin D deficiency occurred in 13% of PID children in whom 25(OH)D concentrations were measured (12/92) and in 22% and 33% of children with predominantly antibody deficiencies and complement deficiencies, respectively, with a median vitamin D supplementation dose of 1000 IU. The median 25(OH)D level was within the range of optimal values in all children: 30.75 ng/mL and 32.7 ng/mL in girls and 30 ng/mL in boys.

**Figure 2:**
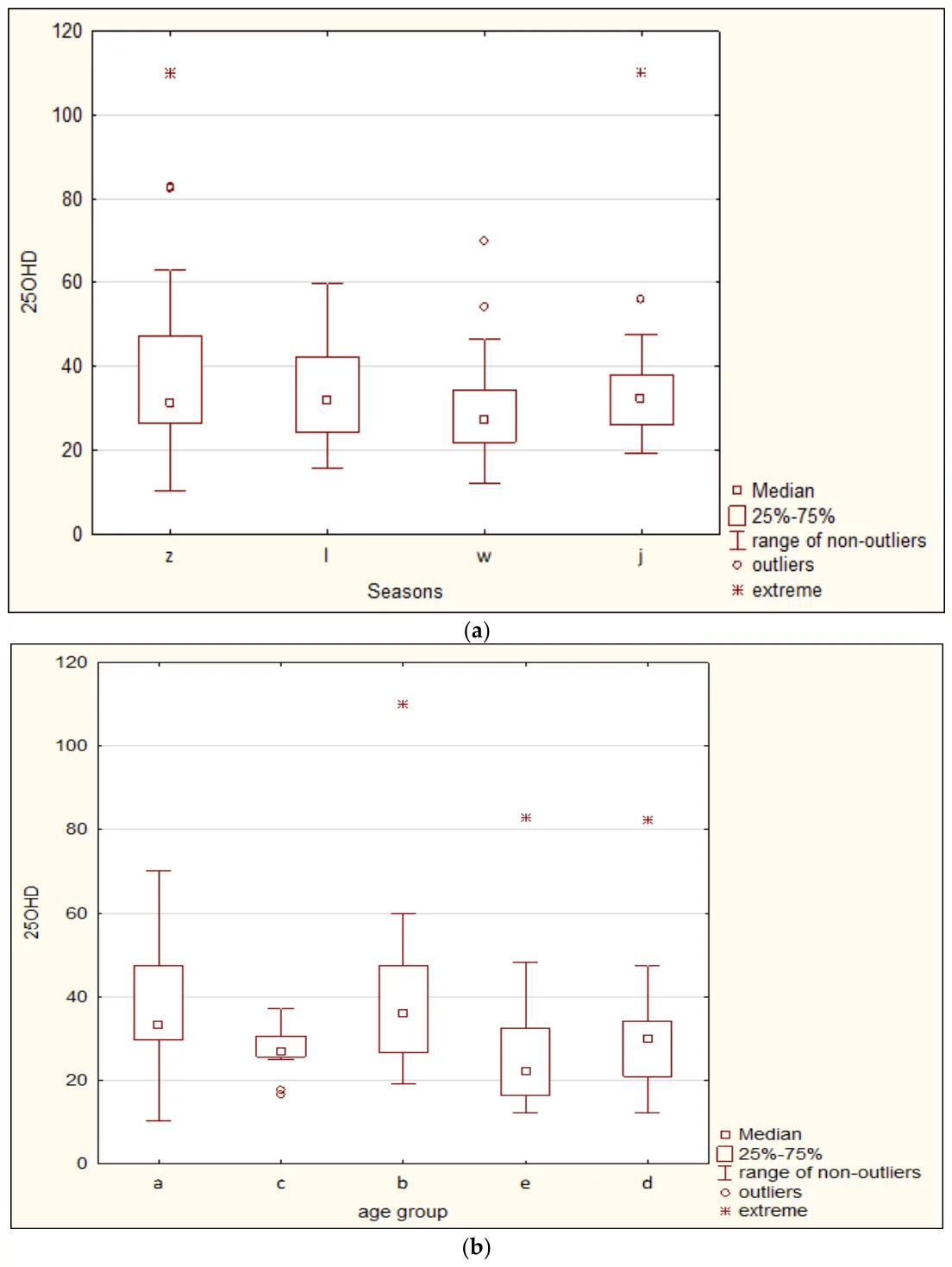
Serum 25(OH) concentrations (ng/mL) according to (**a**) seasons (legend: z–winter; l–summer; w–spring; j–autumn and (**b**) age groups (legend: a: 0–2>; b: 2–6>; c: 6–10>; d: 10–14>; e: 14–18 years).

We found differences in 25(OH)D levels between seasons, with the lowest in spring (median level 27.1 ng/mL), and between age groups, with the lowest in teenagers above 14 years (median 25(OH)D level 22.1 ng/mL); however, no statistical significance was found (Figure 2).

**Figure 3:**
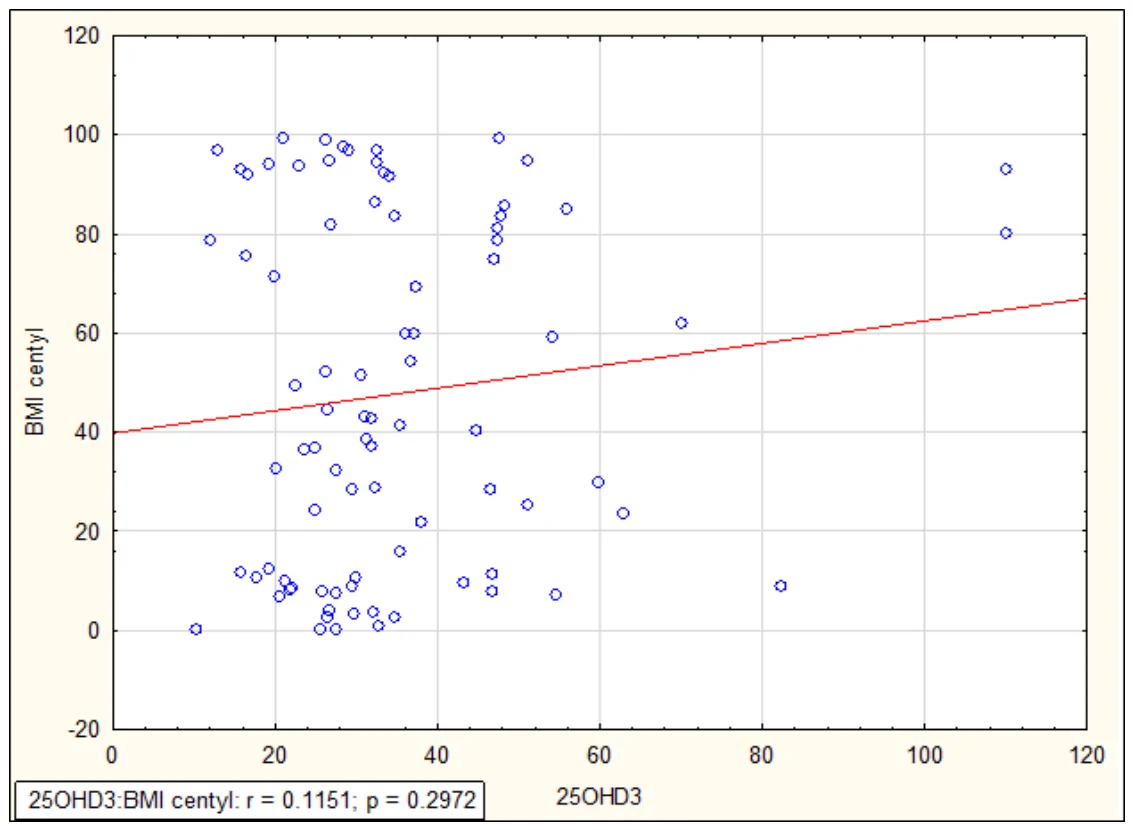
No significant correlation between serum 25(OH)D concentration and BMI percentile (Spearman’s rank correlation).

We found no significant correlation between serum 25(OH)D concentration and BMI percentile (Spearman’s r = 0.1151; *p* = 0.2972) (Figure 3).

The median 25(OH)D level differed among the IUIS groups, with the lowest values, in the suboptimal range, observed in children with predominant antibody and complement deficiencies; however, this difference was not statistically significant, likely limited by the small group sizes (Table 1).

**Figure 4:**
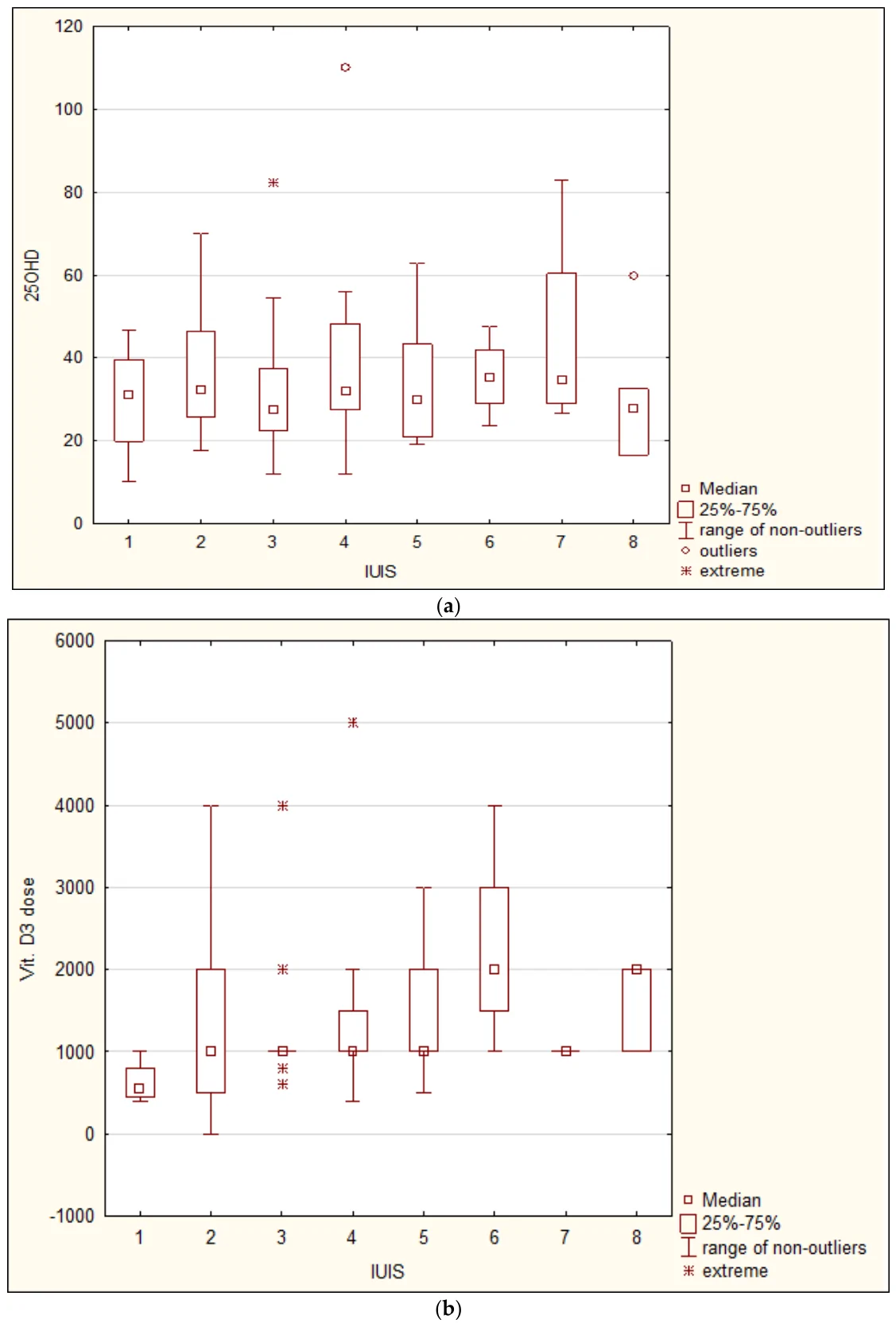
Serum 25(OH)D ng/mL concentration (**a**) and daily vitamin D supplementation dose (**b**) in eight groups according to the IUIS phenotypic classification.

The lowest median 25(OH)D concentration was in children with predominantly antibody deficiencies and complement deficiencies, 27.5 ng/mL and 27.95 ng/mL, respectively (Figure 4a), without statistical significance, which could be attributed to the small sample size in each group. The median daily dose of vitamin D supplementation was 1000 IU in most groups; children from group 1 had a median dose of 550 IU and 2000 IU in group 6 (Figure 4b).

The multiple linear regression analysis showed that serum phosphorus concentration (b = 0.312; *p* = 0.0497) and immunoglobulin G concentration (b = 0.433; *p* = 0.0009) had the strongest associations with serum 25(OH)D concentration among the variables included in the model.

**Figure 5:**
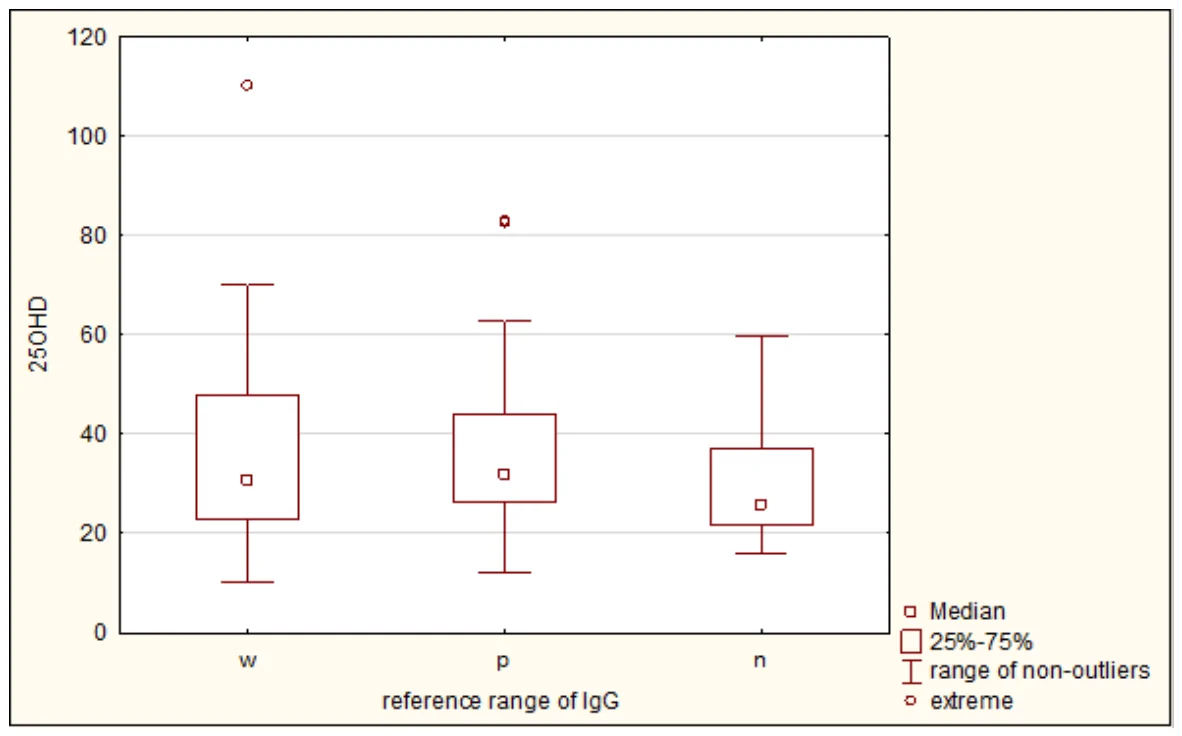
Serum 25(OH)D ng/mL concentrations in children with normal (*p*), high (w) and low (*n*) IgG g/L concentrations for age value without immunoglobulin replacement therapy.

Children without immunoglobulin replacement therapy with low immunoglobulin G levels had lower 25(OH)D concentration, median 27.4 ng/mL; no significant differences were found (Figure 5).

## 4. Discussion

This study showed a normal median concentration of 25(OH)D in children with PID with median dose of vitamin D supplementation 1000 IU. Only 13% of children with PID had 25(OH)D concentrations below the optimal level. These findings suggest that routine vitamin D supplementation at a median dose of 1000 IU/day is generally sufficient to maintain adequate vitamin D status in most pediatric patients with PIDs. This is a significant change compared to our previous study involving 730 children with recurrent respiratory tract infections [23]. This difference may reflect the widespread implementation of routine vitamin D supplementation in accordance with current Polish recommendations [18,19]. As a result, median 25(OH)D concentrations are within the optimal range in most seasons, except spring (Figure 2a). Results also improved in children of all age groups, previously not supplemented with vitamin D after the age of two years. Although adolescents, aged 14 to 18 years, had low median 25(OH)D concentrations (22.1 ng/mL), the values are suboptimal and not in the deficient range (Figure 2b). Adolescents are the age group of children most prone to vitamin D deficiency in general children’s groups. This increased risk is due to higher metabolic demands during growth, reduced adherence to supplementation, lifestyle factors leading to less sun exposure, and dietary insufficiency. Population data show a progressive increase in the prevalence of low vitamin D status with age, with 23% of adolescents having 25(OH)D levels below 20 ng/mL, compared to 12% in children aged 6–11 years and 7% in those aged 1–5 years, as reported by the Endocrine Society in USA guideline [17].

Previous studies have primarily investigated vitamin D deficiency in patients with common variable immunodeficiency (CVID) and X-linked agammaglobulinemia (XLA). Ataxia–telangiectasia syndrome (ATS), DiGeorge syndrome (DGS), Hyper-IgE Syndrome (HIES), and Wiskott–Aldrich syndrome (WAS) [6,7,8,9,10,11,12,24,25,26,27] are analyzed in observational studies, case reports and literature reviews. Vitamin D deficiency, often asymptomatic, is associated with more severe clinical conditions in patients with PID. These groups are the largest among our patients.

Analyzing the concentration of 25(OH)D in PID groups, we found that patients with predominantly antibody deficiencies (group 3) and complement deficiencies (group 8) had lower median 25(OH)D levels, and a higher percentage of patients with suboptimal and deficient values (Table 1, Figure 4a) than other PID children was also noted. The differences were not statistically significant, but the results may be influenced by the small group of patients with extremely rare diseases.

Patients with IgG concentrations below age-specific reference values had lower concentrations of 25(OH)D than those with normal IgG levels, although not statistically significant. This was true for both children receiving immunoglobulin preparations and those not receiving them. The multivariate regression model showed that the strongest association with serum 25(OH)D concentrations was between phosphorus and IgG levels. Our previous study [23] also demonstrated an association between low 25(OH)D concentrations and low IgG. Children with CVID, XLA (X-linked agammaglobulinemia), selective IgA deficiency, and complement deficiency, as well as C2 complement deficiency, have lower serum 25(OH)D levels due to a combination of increased risk for recurrent infections, chronic inflammation, and associated lifestyle and nutritional factors. There are many articles regarding vitamin D deficiency in patients with a predominant antibody deficiency, whereas information on vitamin D status in patients with complement deficiency is lacking. Frequent infections and chronic immune activation in these children increase metabolic demand for vitamin D, which may accelerate its consumption and reduce its storage [28,29]. Additionally, chronic illness often leads to reduced sun exposure and physical activity, as well as dietary insufficiency, all of which are established contributors to low 25(OH)D levels in pediatric populations [30]. Patients with antibody deficiencies like CVID are also more likely to have comorbid conditions (e.g., malabsorption syndromes) that impair vitamin D absorption [31]. Use of immunosuppressive drugs or corticosteroids, sometimes required for autoimmune complications, can interfere with vitamin D metabolism [32]. The cumulative effect of these factors results in a higher prevalence of vitamin D deficiency in children with low IgG levels compared to healthy peers. Frequent infections and ongoing immune activation in these conditions lead to increased utilization and consumption of vitamin D, as vitamin D is required for optimal immune function and regulation of inflammatory pathways [33,34].

In hypogammaglobulinemia, abnormalities in immunoglobulin production are associated with vitamin D deficiency, as demonstrated by proteomic analyses showing that low vitamin D levels correlate with reduced immunoglobulin abundance and altered immune pathways [34]. In congenital complement deficiency, abnormal activation and consumption of complement proteins during infection and inflammation can also be exacerbated by vitamin D deficiency, which impairs complement regulation and further disrupts immune homeostasis [33]. Thus, the interplay of increased metabolic demand, immune dysregulation, and reduced intake or synthesis explains the lower vitamin D levels observed in these patients.

The third largest group among our patients consists of children with diseases of immune dysregulation such as ALPS, XIAP deficiency, STAT3 GOF, CTLA4 haploinsufficiency and APECED. The role of vitamin D is most obvious in APCED, when hypoparathyroidism is diagnosed. Vitamin D deficiency may contribute to autoimmune symptoms in APECED [26,35]. There is no information about vitamin D status in ALPS, XIAP deficiency, STAT3 GOF or CTLA4 haploinsufficiency. Although children are highly prone to vitamin D deficiency due to chronic immune dysregulation, frequent corticosteroid treatment, or systemic inflammation, the median 25OHD level in our group of patients was 32.1 ng/mL, with a median vitamin D supplementation of 1000 IU per day.

Our group of children with autoinflammatory disorders is small, but there are many studies currently evaluating 25-OHD levels. FMF is the most common genetic autoinflammatory disease in the world, and it has been shown that children and adults with FMF have statistically significantly lower vitamin D levels compared to healthy control groups [36]. Studies indicate that the prevalence of severe deficiency during disease flares reaches as high as 70–80% [37]. In interventional studies, patients with FMF were administered high, therapeutic doses of vitamin D3. After six months of normalizing 25(OH)D levels, a marked reduction in the frequency of fever and abdominal pain attacks was observed, along with an improvement in bone mineral density [38]. Maintaining a level above 30 ng/mL (and ideally closer to 40–50 ng/mL) is considered direct, non-pharmacological support for colchicine therapy, helping to suppress disease flares [39]. It has also been shown that children with PFAPA (Periodic Fever, Aphthous Stomatitis, Pharyngitis, and Cervical Adenitis) syndrome exhibit lower serum 25(OH)D concentrations compared to healthy peers [40]. Publications indicate that achieving a level above 30 ng/mL (in some studies calculated as a metabolic threshold of approximately 75 nmol/L) results in a statistically significant reduction of symptoms [41]. However, there are few patients with FMF in our geographical area, and PFAPA syndrome is not included in the IUIS classification, so this group is small and achieved a median 25OHD level of 34.6 ng/mL with a median vitamin D supplementation of 1000 IU per day.

We had no significant difference in median 25(OH)D between girls and boys (32.7 ng/mL vs. 30 ng/mL) but there was a marked male predominance in our children, with a 2.3-fold higher prevalence in boys. This male predominance has also been observed by other researchers, probably because many critical immune genes are located on the X chromosome [42].

Limited data exist regarding the relationship between 25(OH)D status and BMI in patients with PID. There was a negative correlation in ATS patients [9]. We observed no correlation between serum 25(OH)D concentration and BMI expressed as percentiles. This may be explained by the influence of the type of PID on developmental parameters such as height and BMI. Patients with immunodeficiencies affecting cellular and humoral immunity (group 1) had the lowest BMI, while those with combined immunodeficiencies associated with syndromic features (group 2) exhibited the lowest height.

The strengths of this study include a relatively large cohort of pediatric patients representing multiple IUIS diagnostic categories and the evaluation of vitamin D status under routine clinical practice. However, several limitations should be acknowledged.

First, the retrospective cross-sectional design precludes conclusions regarding causality. Second, serum 25(OH)D concentrations were available for only a subset of patients. Third, vitamin D supplementation was assessed from medical records and parental reports rather than by objective measures of adherence. Finally, several potentially important determinants of vitamin D status, including dietary intake, sunlight exposure, physical activity, corticosteroid treatment, and gastrointestinal malabsorption, were not systematically evaluated.

Our research shows that the empirical dose of vitamin D recommended by the Polish Society of Pediatric Endocrinology and Diabetes and the Expert Panel [18,19] is sufficient in most children with PID. Nevertheless, additional prospective multicenter studies are warranted to determine whether individualized supplementation strategies should be considered for selected diagnostic subgroups, particularly those with predominantly antibody deficiencies and complement deficiencies.

## 5. Conclusions

In this retrospective cross-sectional study, children with PIDs receiving routine vitamin D supplementation achieved a median serum 25(OH)D concentration within the recommended range, whereas vitamin D deficiency was identified in only 13% of patients with available laboratory measurements. Serum phosphorus and IgG concentrations were independently associated with serum 25(OH)D levels, suggesting a potential relationship between vitamin D status and immune function that warrants further investigation.

Overall, our findings indicate that the vitamin D supplementation regimen currently recommended for the general pediatric population appears to be sufficient for maintaining adequate vitamin D status in most children with primary immunodeficiency disorders. Self-reported supplementation is a major limitation with high potential for measurement error and could have significant implications for the interpretation of the results because all patients self-administered vitamin D once a day.

## Figures and Tables

**Table 1:** Serum 25(OH)D concentration, vitamin D supplementation dose, BMI percentile, and height percentile according to the IUIS phenotypic classification.

Variables	All	Group 1	Group 2	Group 3	Group 4	Group 5	Group 6	Group 7	Group 8
25(OH)D (ng/mL) median, range	30.75 10.3–110	31.1 10.3–46.6	32.2 17.7–70	27.5 12.1–82.3	32.1 12.1–110	30 20.9–62.9	35.35 23.6–47.5	34.6 26.6–82.8	27.95 16.55–59.8
25(OH)D deficiency (%)	13	25	4	22	6	14	0	0	33
Vitamin D dose (IU) median, range	1000 0–5000	550 400–1000	1000 0–4000	1000 400–2000	1000 400–5000	1000 500–3000	2000 1000–4000	1000	2000 1000–2000
BMI (percentile) median, range	38.7 0.1–99.4	8.9 0.2–11.3	28.5 0.1–94.7	60 0.1–97.9	80.3 0.1–97.7	32.8 9.7–99.3	48.3 2.6–99.4	21.8 2.1–57.3	52.75 3.1–94.6
High (percentile) median, range	19.8 0.1–99.7	49.9 0.1–96	5.4 0.1–89.2	66 0.1–99.7	36.55 3.7–99.1	24.7 2.7–96.5	44.25 0.4–95.7	19.8 14–72	24.2 8.3–43.5

## Data Availability

Data are not available for privacy reasons.

## Supplementary Materials

The following supporting information can be downloaded at: https://www.mdpi.com/article/10.3390/nu18172845/s1, Table S1: Summary table presenting the distribution of diagnoses according to the IUIS classification.

## Abbreviations

The following abbreviations are used in this manuscript:

PID	primary immunodeficiency disorders
IEI	inborn errors of immunity
ATS	ataxia telangiectasia syndrome
CVID	common variable immunodeficiency
ESID	European Society for Immunodeficiencies
IUIS	International Union of Immunological Societies
ALPS	autoimmune lymphoproliferative syndrome
APDS	activated p110δ syndrome
APS-1	autoimmune polyendocrine syndrome type 1
APECED	autoimmune polyendocrinopathy with candidiasis and ectodermal dystrophy
CARD11	caspase recruitment domain-containing protein 11
CGD	chronic granulomatous disease
CHARGE	Coloboma, Heart defects, Atresia choanae, Retardation, Genital abnormalities, Ear
CHH	cartilage hair hypoplasia
CTLA4	cytotoxic T-lymphocyte associated protein 4
DGS	DiGeorge syndrome
FCAS	familial cold autoinflammatory syndrome
FMF	familial Mediterranean fever
GOF	gain of function
HIES	Hyper-IgE syndrome
ICF2	immunodeficiency with centromeric instability and facial anomalies type 2
IFNGR1	interferon gamma receptor 1 deficiency
IGLL1	immunoglobulin lambda like polypeptide 1
JAK-STAT	Janus kinase-signal transducer and activator of transcription
mTOR	mechanistic target of rapamycin
MVK	mevalonate kinase deficiency
SCID	severe combined immunodeficiency
STAT3	signal transducer and activator of transcription 3
THI	transient hypogammaglobulinemia of infancy
TRAPS	TNF receptor-associated periodic syndrome
WAS	Wiskott-Aldrich syndrome
XIAP	X-linked inhibitor of apoptosis
XLA	X-linked agammaglobulinemia
