# Supplementary material for: Vitamin D Status in Children with Primary Immunodeficiency Disorders Receiving Vitamin D Supplementation: A Retrospective Cross-Sectional Study
Source: Nutrients. 2026 Aug 31;18(17):2845. doi: 10.3390/nu18172845 (PMC13567533; doi:10.3390/nu18172845)
Supplement: Supplementary file 1 [file nutrients-18-02845-s001.zip › nutrients-4406536-supplementary.pdf]

Table S1. A summary table presenting the distribution of diagnoses according to the IUIS classification

PID diagnosis	Number of patients
Group 1	5
SCID (severe combined immunodeficiency)	5
Group 2	34
ATS (Ataxia telangiectasia syndrome)	2
CHARGE (Coloboma, Heart defects, Atresia choanae, Retardation, Genital abnormalities, Ear)	1
CHH (cartilage hair hypoplasia)	2
DGS (DiGeorge syndrome)	15
ICF2 (immunodeficiency with centromeric instability and facial anomalies type 2)	1
Nijmegen syndrome	8
ORAI1 deficiency	2
Roifman syndrome	2
WAS (Wiskott-Aldrich syndrome)	1
Group 3	40
CVID (Common variable immunodeficiency)	5
Selective IgA deficiency	9
Isolated IgG subclass deficiency	7
Selective IgM deficiency	1
THI (transient hypogammaglobulinemia of infancy)	9
XLA (X-linked agammaglobulinemia)	3
IGLL1 (immunoglobulin lambda like polypeptide 1)	1
IKAROS deficiency	2
APDS (Activated p110 syndrome)	2
	1

CARD11 GOF (caspase recruitment domain-containing protein 11, gain of function)	
Group 4<br>ALPS (Autoimmune lymphoproliferative syndrome)<br>ALPS-like<br>XIAP (X-linked inhibitor of apoptosis) deficiency<br>STAT3 GOF (signal transducer and activator of transcription 3, gain of function)<br>CTLA4 (cytotoxic T-lymphocyte associated protein 4) haploinsufficiency<br>APS-1 (autoimmune polyendocrine syndrome type 1)	22<br>9<br>5<br>4<br>2<br>1<br>1
Group 5<br>CGD (chronic granulomatous disease)<br>Sever congenital neutropenia 1	13<br>11<br>2
Group 6<br>IFNGR1 (interferon gamma receptor 1) deficiency	4<br>4
Group 7<br>TRAPS (TNF receptor-associated periodic syndrome)<br>FMF (familial Mediterranean fever)<br>MVK (mevalonate kinase deficiency)<br>FCAS (familial cold autoinflammatory syndrome)	6<br>3<br>1<br>1<br>1
Group 8<br>Complement deficiency	8<br>8
